# Shear stress induces endothelial-to-mesenchymal transition via the transcription factor Snail

**DOI:** 10.1038/s41598-017-03532-z

**Published:** 2017-06-13

**Authors:** Marwa M. Mahmoud, Jovana Serbanovic-Canic, Shuang Feng, Celine Souilhol, Rouyu Xing, Sarah Hsiao, Akiko Mammoto, Jing Chen, Markus Ariaans, Sheila E. Francis, Kim Van der Heiden, Victoria Ridger, Paul C. Evans

**Affiliations:** 1 Department of Infection, Immunity and Cardiovascular Disease, the INSIGNEO Institute for In Silico Medicine and the Bateson Centre, University of Sheffield, England, UK; 2 ERASMUS MC, Rotterdam, The Netherlands; 3 Vascular Biology Program, Department of Surgery, Boston, MA USA; 4 Department of Ophthalmology, Boston Children’s Hospital, Harvard Medical School, Boston, MA USA

## Abstract

Blood flow influences atherosclerosis by generating wall shear stress, which alters endothelial cell (EC) physiology. Low shear stress induces dedifferentiation of EC through a process termed endothelial-to-mesenchymal transition (EndMT). The mechanisms underlying shear stress-regulation of EndMT are uncertain. Here we investigated the role of the transcription factor Snail in low shear stress-induced EndMT. Studies of cultured EC exposed to flow revealed that low shear stress induced Snail expression. Using gene silencing it was demonstrated that Snail positively regulated the expression of EndMT markers (Slug, N-cadherin, α-SMA) in EC exposed to low shear stress. Gene silencing also revealed that Snail enhanced the permeability of endothelial monolayers to macromolecules by promoting EC proliferation and migration. *En face* staining of the murine aorta or carotid arteries modified with flow-altering cuffs demonstrated that Snail was expressed preferentially at low shear stress sites that are predisposed to atherosclerosis. Snail was also expressed in EC overlying atherosclerotic plaques in coronary arteries from patients with ischemic heart disease implying a role in human arterial disease. We conclude that Snail is an essential driver of EndMT under low shear stress conditions and may promote early atherogenesis by enhancing vascular permeability.

## Introduction

Endothelial-to-mesenchymal transition (EndMT) is characterised by multiple morphological and physiological changes including loss of cell polarity, disruption of intercellular junctions, increased proliferation, delamination and migration of cells into surrounding tissue^1, 2^. This process is essential for atrioventricular valve formation where EC expressing mesenchymal markers invade the cardiac jelly to form a cardiac cushion which subsequently forms the valves^3^. Recent studies have also implicated EndMT in the pathophysiology of vascular diseases including cerebral cavernous malformations^4^, pulmonary hypertension^5^, vascular graft remodelling^6^ and atherosclerosis^7–10^.

Wall shear stress, imposed on the vessel lumen by the flow of blood, controls the localization of atherosclerotic lesions by regulating fundamental physiological activities in EC^11^. Low shear stress at branches and bends of arteries promotes atherosclerosis by inducing endothelial injury and proliferation^12–15^, which enhances permeability to cholesterol-rich lipoproteins^16^. By contrast, high shear stress protects arteries by inducing a quiescent, protected state in EC^17, 18^. The endothelium at disease-prone sites constitutes a highly heterogeneous population of cells that display multiple pathophysiological features including inflammatory activation, proliferation, apoptosis and senescence^11–15^. Notably, recent studies revealed that low shear stress can also promote EndMT^7–9, 19^. Moreover, EC with mesenchymal characteristics were identified overlying atherosclerotic plaques suggesting that EndMT may contribute to atherogenesis^7, 8^. The signaling intermediaries that link mechanical forces to EndMT are only partially understood. Moonen *et al*. found that laminar shear stress protects EC from mesenchymal transition via activation of MEK5/ERK5 signalling which activates the transcription factors KLF4 and KLF2^8^. On the other hand, we recently demonstrated that low shear stress promotes EndMT via activation of the transcription factors GATA4 and TWIST1^9^. However, the mechanisms that promote EndMT downstream from TWIST1 in sheared EC remain uncertain and are an important focus of the current study. We assessed whether the transcription factor Snail fulfills this role because it is essential for epithelial-to-mesenchymal transition (EMT) during development^20–22^, but its role in mechanical EndMT has not previously been studied. Here we used cultured EC exposed to flow and animal models with varying levels of flow to demonstrate that Snail plays an essential role in the induction of EndMT in response to disturbed flow.

## Material and Methods

### Study approval

For studies of human cells, experiments were approved by the University of Sheffield Research Ethics Committee (reference SMBRER310) and all subjects gave informed consent. Studies using human cells and tissues were used in accordance to the standards set by the Declaration of Helsinki. For animal studies, all procedures were approved by the University of Sheffield ethics committee and performed in accordance with the UK Home Office Animals (Scientific Procedures) Act 1986 and in accordance with Directive 2010/63/EU of the European Parliament on the protection of animals used for scientific purposes.

### Antibodies and reagents

Antibodies targeting human and murine Snail (ab180714, Abcam), human N-cadherin (ab12221, Abcam), human VE-cadherin (555661, BDPharmingen), murine CD31 (102514, Biolegend) and Ki67 (ab15580, Abcam) were obtained commercially. AlexaFluor-conjugated secondary antibodies and TO-PRO-3 were from Invitrogen.

### Isolation of EC from porcine aortae

Pig aortas from 4–6 month old animals (approximately 80 kg) were obtained immediately after slaughter from a local abattoir. They were cut longitudinally along the outer curvature to expose the lumen. EC exposed to high (outer curvature) or low (inner curvature) shear stress were harvested using collagenase (1 mg/ml for 10 minutes at room temperature) prior to gentle scraping. RNA was extracted using an RNeasy MiniKit (Qiagen) and concentrated using an RNeasy MinElute Cleanup kit (Qiagen) and the purity and integrity of total RNA samples was assessed using a Bioanalyser (Agilent).

### EC culture and exposure to shear stress

Human umbilical vein EC (HUVEC) and porcine aortic EC (PAEC) were isolated using collagenase digestion and cultured. The purity of HUVEC was assessed by measuring the expression of VE-cadherin which was typically observed in >99% cells (Supplementary Fig. 1). It was confirmed that collagenase-isolated PAEC expressed PECAM-1 and had negligible expression of α-smooth muscle actin (SMA) or CD14 (monocyte/macrophage marker)^15^. EC at passage 3–5 were cultured until confluent in 6 well plates and exposed to flow using an orbital shaking platform (PSU-10i; Grant Instruments) housed inside a cell culture incubator^9, 13, 23^. The radius of orbit of the orbital shaker was 10 mm and the rotation rate was set to 210 rpm. Previous computational fluid dynamic analysis revealed that these conditions generate low shear stress (approximately 5 dyn/cm^2^) with rapid variations in direction in the centre and high shear stress (approximately 15 dyn/cm^2^) with relatively uniform direction at the periphery^13, 23^. In some experiments, cells were harvested by scraping from specific regions of the well using a standardized template to identify the high and low shear stress regions. Alternatively, EC were cultured on Ibidi gelatin-coated µ-Slides (Ibidi GmbH) until they reached confluency and exposed to low unidirectional (4 dyn/cm^2^), low oscillatory (+/−4 dyn/cm^2^, 0.5 Hz) or high unidirectional (13 dyn/cm^2^) shear stress using the Ibidi pump system^9^.

### Flow cytometry

Adherent endothelial cells were detached from culture flasks with trypsin, washed and resuspended in PBS containing 7% foetal calf serum. Cells were then incubated with anti-VE-cadherin antibodies conjugated to PeCy7 (clone 16B1, eBioscience) or IgG1K-PeCy7 (eBioscience) for 30 min at 4 °C. After 2 washes, fluorescence was measured using a LSRII BD Bioscience flow cytometer.

### RNA interference

Gene silencing was performed using siRNA against Snail1 (OnTargetPlusSMARTPOOL, L-010847-01, Dharmacon) using the Lipofectamine RNAiMAX transfection system as described^9^. Non-targeting scrambled sequences were used as a control (D-001810-01-50 ON-TARGETplus Non targeting siRNA#1, Dharmacon).

### Comparative real time PCR

The levels of human or porcine transcripts were assessed by quantitative real time PCR (qRT-PCR) using gene-specific primers as described^9, 15^. Relative gene expression was calculated by comparing the number of thermal cycles that were necessary to generate threshold amounts of product. Fold changes were calculated using the ΔΔCt method.

### Immunofluorescent staining of cultured EC

The expression levels of proteins were assessed by immunofluorescent staining using antibodies against Snail, N-cadherin or Ki67 and AlexaFluor488- or Alexafluor568-conjugated secondary antibodies. Nuclei were identified using DAPI (Sigma). Image analysis was performed using Image J software (1.49p) to calculate average fluorescence. Isotype controls or omission of the primary antibody was used to control for non-specific staining.

### *In vitro* migration assay

Wounds were created on confluent EC monolayers using a pipette tip and migration of EC into the wounded area was visualised using time-lapse confocal microscopy (Leica AF6000 Timelapse microscope) as described^24^. Images were captured at 3 min intervals for 20 h after wounding. The distance migrated was determined by measuring the position of the monolayer edge in relation to the starting position using LAS_AF Software.

### Assay of permeability

The permeability of EC monolayers exposed to flow was determined using rhodamine-labelled albumin as described^9^. HUVEC were cultured in Transwell inserts and then exposed to orbital shaking for 72 h or to static conditions as a control. The media in the upper compartment was then replaced with 10% serum-supplemented DMEM containing 1% BPA and rhodamine-labeled albumin (1 mg/ml). Media in the lower compartment was sampled at 1 h and fluorescence was measured using a fluorimeter (Varioskan, Thermoscientific) with excitation at 570 nm and emission at 600 nm.

### Mice

Mice were housed under specific-pathogen free conditions. Mice with EC deletion of *Twist1* (*Tie2-Twist1* ^*KO*^) were generated by crossing Tie2-Cre expressing mice (Jackson Laboratory stock #004128) with *Twist1* floxed mice (*Twist1* ^*flox|flox*^)^25^. Constrictive cuffs were applied to the right carotid artery of isofluorane-anaesthetized mice following published methods^26^. Both halves of the cast are placed around the common carotid artery and secured with sutures. To minimise vascular injury the diameter of the internal groove of the cuff was carefully chosen to generate a stenosis of 75%. After 2 weeks mice were killed using Pentobarb (200 mg/kg i.p.). The expression levels of specific proteins were assessed in EC by *en face* staining as described^9, 13, 15^.

### Histology

Coronary arteries from patients undergoing cardiac transplantation for ischaemic heart disease or dilated cardiomyopathy were obtained under the Sheffield Teaching Hospitals Institutional Review Board authorisation STH16346 (12/NW/0036). Sections made from formalin-fixed, paraffin-embedded tissues were incubated in xylene for 10 min, hydrated by sequential exposure to decreasing concentrations of ethanol (100% to 50%) and water. Heat-mediated antigen retrieval was carried out in tris-sodium citrate in a heated water bath. The sections were then blocked for endogenous peroxidase activity and incubated in 1% milk prior to overnight incubation with primary antibodies followed by HRP-conjugated secondary antibodies and substrate (VECTASTAIN Elite ABC kit; Vector laboratories and Dako). Sections were then counterstained with hematoxylin and visualized by bright field microscopy.

### Statistics

Differences between samples were analysed using an unpaired or paired Student’s t-test or ANOVA (*p < 0.05, **p < 0.01, ***p < 0.001).

### Data availability statement

All data generated or analysed during this study are included in this published article (and its Supplementary Information files).

## Results

### Low shear stress promoted endothelial expression of mesenchymal genes via Snail

**Figure 1:**
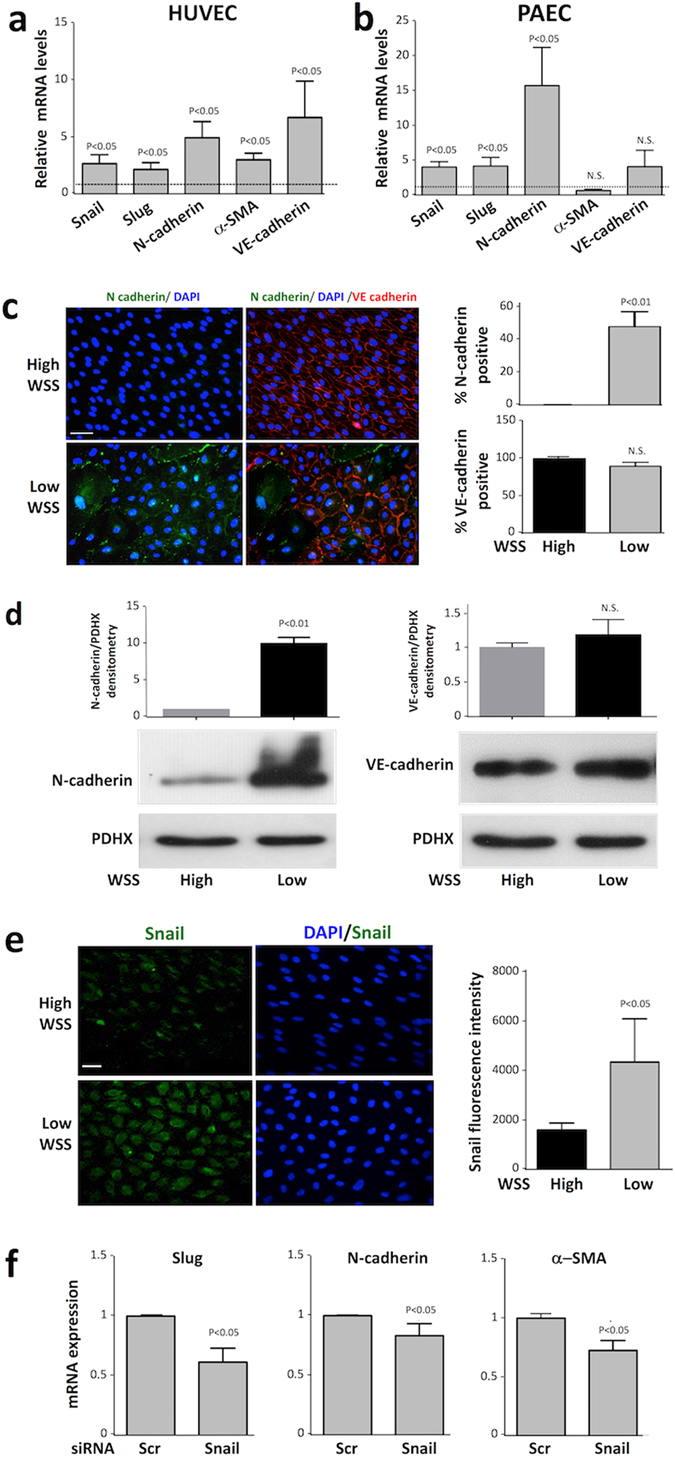
Low shear stress induced mesenchymal genes via Snail. (**a**) HUVEC were exposed to low oscillatory (+/−4 dyn/cm^2^) or high (13 dyn/cm^2^) wall shear stress (WSS) using a parallel plate system. (**b**) PAEC were exposed to orbital flow to generate low (5 dyn/cm^2^) or high (15 dyn/cm^2^) wall shear stress. (**a**,**b**) After 72 h, levels of EndMT marker transcripts and VE-cadherin transcripts were quantified by qRT-PCR. The expression level at the low WSS site is presented relative to the expression at the high WSS site (normalised to 1; dotted line). Data were pooled from six independent experiments using cells from different donors and mean levels +/− SEM are shown. (**c–e**) HUVEC were exposed to orbital flow to generate low (5 dyn/cm^2^) or high (15 dyn/cm^2^) WSS for 72 h. (**c**) Expression of N-cadherin (green) and VE-cadherin (red) was determined by immunofluorescent staining and co-staining using DAPI (blue). Scale bar, 50 μm. The proportion of cells that expressed N-cadherin or VE-cadherin was measured. (**d**) The expression levels of N-cadherin (left) and VE-cadherin (right) were assessed by Western blotting using specific antibodies and anti-PDHX antibodies were used to control for total protein levels. Representative blots are shown. Bands were quantified by densitometry. (**e**) Expression of Snail (green) was determined by immunofluorescent staining and co-staining using DAPI (blue). Scale bar, 50 μm. Fluorescence intensity was quantified in multiple cells. (**f**) HUVEC were transfected with siRNA targeting Snail or with scrambled sequences and exposed to orbital flow for 72 h. Cells exposed to low WSS (5 dyn/cm^2^) were collected and transcript levels of Slug, N cadherin and α-SMA were quantified by qRT-PCR. (**c**–**f**) Data were pooled from three independent experiments using cells from different donors and mean levels +/− SEM are shown.

To determine whether shear stress regulates the expression of mesenchymal genes we exposed cultured EC to flow. qPCR revealed that the expression of several mesenchymal markers (Snail, Slug, N-cadherin and αSMA) was elevated in HUVEC exposed to low, oscillatory shear compared to high shear stress using a parallel plate apparatus (Fig. 1a). Similarly, PAEC exposed to low shear using an orbital system expressed higher levels of Snail, Slug and N-cadherin compared to cells under high shear (Fig. 1b). Thus we conclude that low shear stress promotes mesenchymal marker expression in cultured EC. Similarly, immunofluorescent staining (Fig. 1c) and Western blotting (Fig. 1d) revealed that N-cadherin protein was expressed in a proportion of cells exposed to low shear stress but not in cells exposed to high shear.

A full EndMT involves reduced expression of VE-cadherin, a component of EC adherens junctions that plays a central role in vascular homeostasis^27^. We measured the expression of VE-cadherin in cultured EC exposed to flow using a combination of qRT-PCR, immunofluorescent staining and Western blotting. Interestingly, qRT-PCR revealed that VE-cadherin mRNA levels were enhanced in HUVEC exposed to low shear compared to high shear conditions (Fig. 1a). However low shear stress did not induce VE-cadherin mRNA in PAEC (Fig. 1b), a finding which may relate to differences between arterial and venous responses to flow. Moreover, Western blotting (Fig. 1d) and immunofluorescent staining (Fig. 1c) demonstrated that VE-cadherin protein levels in HUVEC were unaltered by shear (apart from the loss of VE-cadherin from occasional cells; Fig. 1c). Collectively, these data lead us to conclude that VE-cadherin expression was not reduced in EC exposed to low shear stress despite their acquisition of mesenchymal markers suggesting that the EC transition towards a mesenchymal state was partial.

We tested the hypothesis that Snail regulates mesenchymal genes under low shear stress. Consistent with this, immunofluorescent staining revealed that Snail protein expression was significantly higher in EC exposed to low compared to high shear stress (Fig. 1e). The function of Snail was studied using Snail-targeting siRNA that reduced Snail mRNA and protein expression (Supplementary Fig. 2). Silencing of Snail significantly reduced the expression of Slug, αSMA and N-cadherin in EC exposed to low shear stress (Fig. 1f). Thus we conclude that Snail drives EndMT in response to low shear stress by inducing mesenchymal genes.

### Snail promoted migration, proliferation and permeability of EC

**Figure 2:**
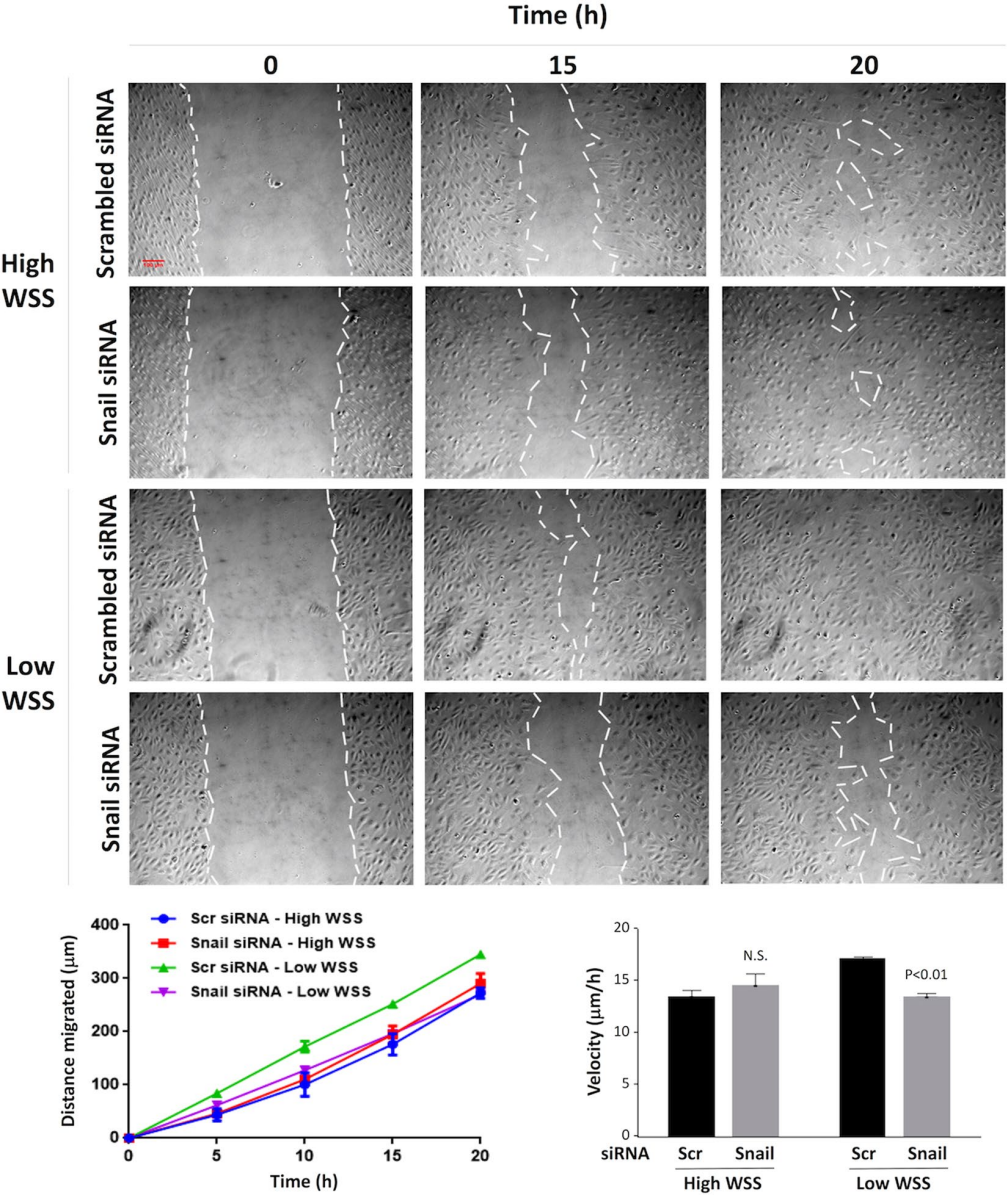
Snail enhanced migration under low shear stress. HUVEC were treated with siRNA targeting Snail, or with scrambled non-targeting siRNA (or were untreated as a control). They were then cultured in 6 well plates prior to exposure to low or high wall shear stress (WSS) for 72 h using an orbital plate system. To assess cell migration, a scratch wound was made in the monolayer, and cells were imaged for 20 h. Representative images are shown (scale bar 100 μm). The distance migrated at multiple time points (lower left) and average velocity (lower right) was determined and mean values +/− SEM are shown. Data were pooled from four independent experiments using cells from different donors and mean levels +/− SEM are shown. Differences between means were assessed using a two-way ANOVA.

Since Snail was regulated by flow, we studied its function in sheared endothelium by gene silencing. Firstly, we studied the role of Snail in EC migration because this function is a key characteristic of EndMT. Scratch assays revealed that EC pre-conditioned under low shear stress migrated with higher velocity than cells pre-exposed to high shear (Supplementary Fig. 3a). Silencing of Snail significantly reduced migration of cells exposed to low shear stress (Fig. 2, lower panels) but did not influence cells exposed to high shear (Fig. 2, upper panels) or static conditions (Supplementary Fig. 3b).

**Figure 3:**
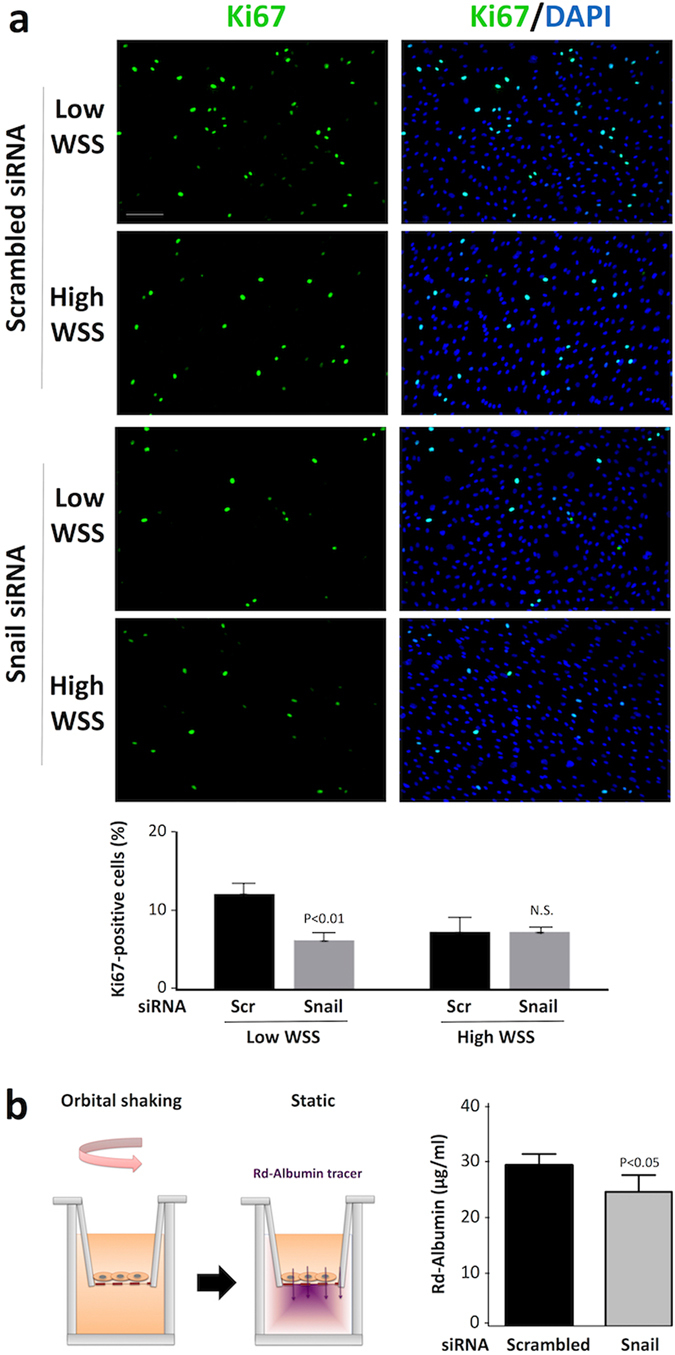
Snail enhanced proliferation and permeability in EC exposed to low shear stress. HUVEC were treated with siRNA targeting Snail or with scrambled (Scr) non-targeting siRNA. (**a**) Cells were subsequently cultured in 6 well plates prior to exposure to orbital flow to generate low or high wall shear stress (WSS) for 72 h. Cell proliferation was quantified by immunofluorescent staining using anti-Ki67 antibodies and co-staining using DAPI. Representative images are shown (Scale bar, 50 μm). The % Ki67-positive cells were calculated for multiple fields of view in four independent experiments using cells from different donors and mean levels +/− SEM are shown. Differences between means were assessed using a 2-way ANOVA. (**b**) Cells cultured on Transwell inserts were exposed to orbital flow (low WSS) for 72 h prior to assessment of endothelial permeability under static conditions using rhodamine (Rd)-albumin as a tracer. A schematic is shown (left panel). The concentration of Rd-albumin in the lower compartment was measured. Data were pooled from four independent experiments using cells from different donors and mean values +/− SEM are shown (right panel). Differences between means were assessed using an unpaired t-test.

We next studied the influence of Snail on cell division and observed that the rate of proliferation of EC exposed to low shear stress was significantly reduced by silencing of Snail (Fig. 3a), indicating that Snail is a positive regulator of this process. By contrast, silencing of Snail did not influence proliferation of EC exposed to high shear (Fig. 3a) or static conditions (Supplementary Fig. 4). The permeability of endothelial monolayers to macromolecules was also studied because this is tightly linked to mitosis rate^16^. Endothelial permeability was tested by culturing EC monolayers on transwell inserts prior to their exposure to low shear stress using an orbital platform and assessment of rhodamine-albumin transport from the upper to the lower chamber (Fig. 3b, left panel). EC permeability was reduced by silencing of Snail (Fig. 3b, right panel) indicating that Snail promotes permeability to macromolecules in low shear conditions. By contrast, silencing of Snail did not influence permeability in HUVEC monolayers under static conditions (Supplementary Fig. 5).

Thus it was concluded that Snail influenced the physiological properties of EC exposed to low shear stress by promoting EC migration, proliferation and increased permeability to macromolecules, but it did not influence cells exposed to high shear stress or static conditions.

### Low shear stress induced Snail expression at atherosusceptible sites

**Figure 4:**
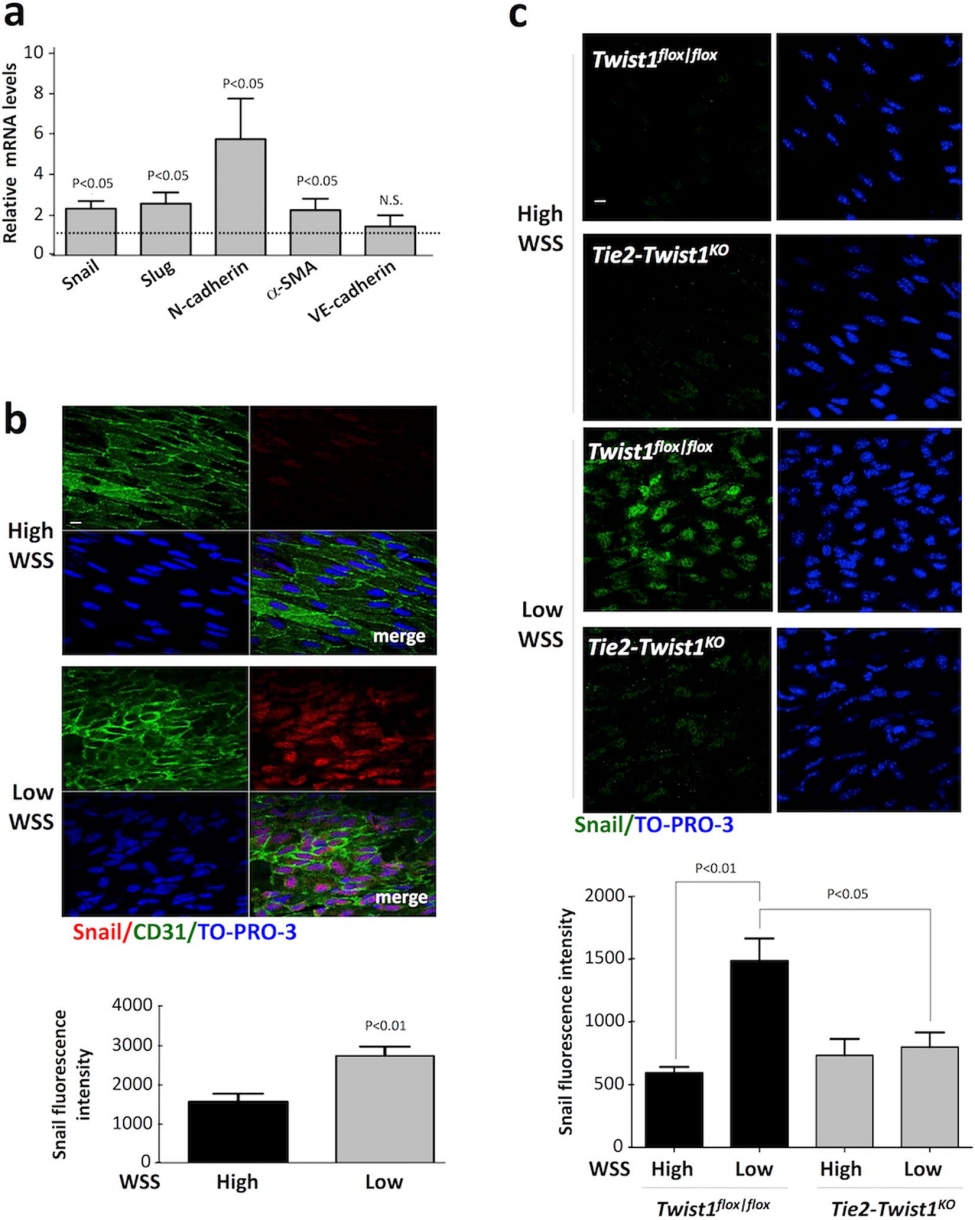
Snail was preferentially expressed at low shear atherosusceptible sites. (**a**) EC were freshly-isolated from low wall shear stress (WSS; inner curvature) and high WSS (outer curvature) regions of the aorta in six pigs. Levels of Snail, Slug, N-cadherin, α-SMA and VE-cadherin mRNA were quantified by qRT-PCR. The expression level at the low WSS site is presented relative to the expression at the high WSS site (normalised to 1; dotted line). Mean levels +/− SEM are shown. (**b**,**c**) EC at low WSS (susceptible) or high WSS (protected) regions of the aorta were studied by *en face* staining. (**b**) C57BL/6 mice (n = 5) were stained using anti-Snail antibodies (red), co-stained using anti-CD31 antibodies (green) and counterstained using TO-PRO-3 (DNA; blue). (**c**) TWIST1^cKO^ or TWIST1^fl/fl^ mice (n = 4 each group) were stained using anti-Snail antibodies (green) and counterstained using TO-PRO-3 (DNA; blue). Representative images (scale bar, 10 μm) and quantitation of Snail fluorescence levels (mean +/− SEM) are shown. Differences between means were assessed using a paired t-test (**b**) or two-way ANOVA (**c**).

Since Snail was induced by low shear stress *in vitro*, we predicted that it would be expressed at atherosusceptible sites in the aorta. Consistent with this, qPCR analysis revealed that the expression of Snail and several of its downstream targets (Slug, N-cadherin, αSMA) was elevated in EC freshly-isolated from a low shear (inner curvature) region compared to those freshly-isolated from a high shear region (outer curvature) of the porcine aorta (Fig. 4A). By contrast, the expression of VE-cadherin was similar at low and high shear stress regions (Fig. 4a). *En face* staining revealed that Snail protein expression was enriched at a low shear stress site in the murine aorta (Fig. 4b). It is notable that Snail localized to the nucleus of EC at the low shear stress site suggesting that it is active under these mechanical conditions. We have previously demonstrated that Twist1 promotes EndMT and lesion development at low shear stress sites^9^. To assess the role of Twist1 in the regulation of Snail *in vivo* we deleted Twist1 from EC by crossing floxed mice (Twist1^flox/flox^) with Tie2-Cre transgenics (knockout mice are referred to as Tie2-Twist1^KO^). *En face* staining revealed that endothelial expression of Snail at the low shear stress site was reduced in Tie2-Twist1^KO^ compared to Twist1^flox/flox^ mice (Fig. 4c), indicating that Twist1 positively regulates Snail in atherosusceptible endothelium.

**Figure 5:**
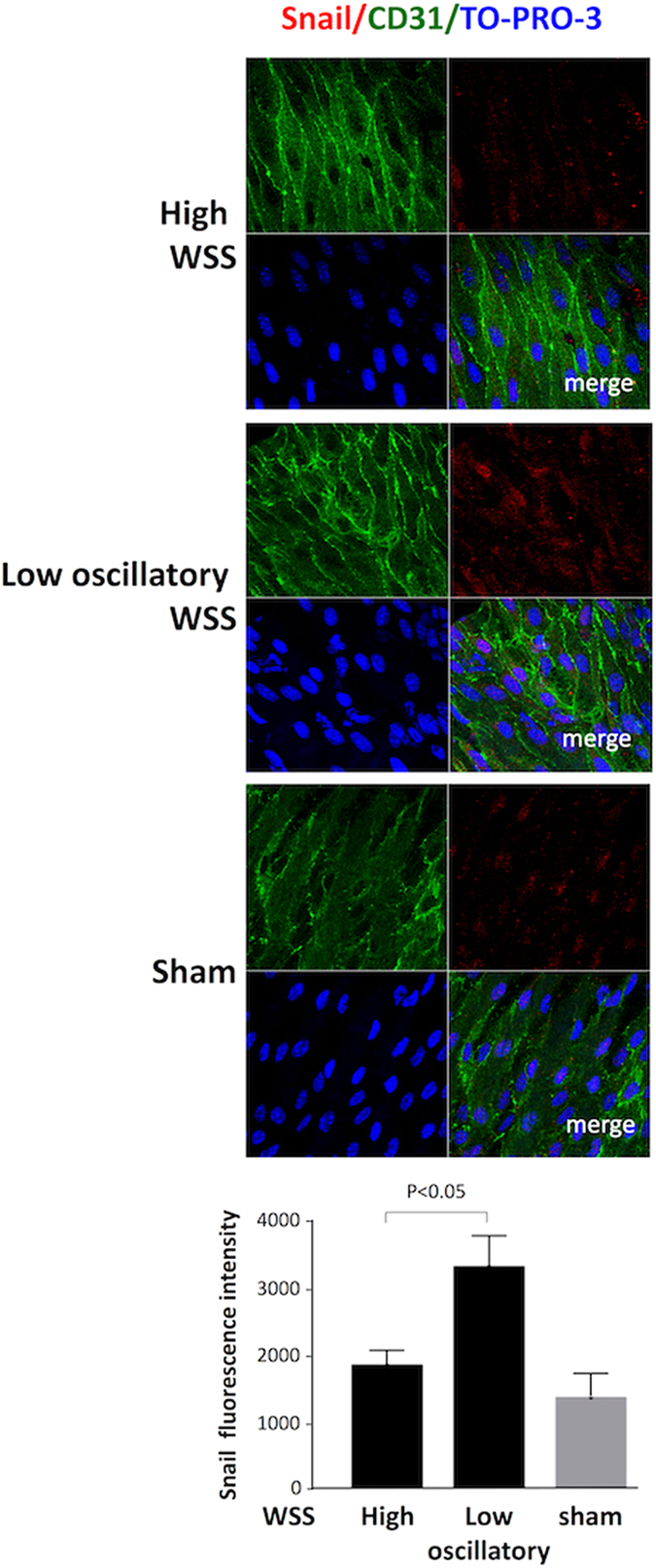
Snail was induced by low oscillatory shear stress in murine carotid arteries. Flow-altering, constrictive cuffs were placed on the right carotid arteries of C57BL/6 mice. They generated anatomically distinct regions exposed to high or low oscillatory wall shear stress (WSS; as indicated). Contralateral arteries were used as an experimental control. Experimental arteries were harvested after 14 days and *en face* staining was performed using anti-Snail antibodies (red), anti-CD31 antibodies (green) and the nuclear counter stain TO-PRO-3 (blue). Representative images (scale bar, 10 μm) and quantitation of Snail expression (n = 3; mean +/− SEM) are shown.

Atherosusceptible regions of arteries are associated with altered mass transport, inflammation and other physiological features as well as low shear stress. Therefore, to examine directly whether altered shear stress can induce Snail expression *in vivo* we modified flow in the murine carotid artery using a constrictive cuff. This causes tapering of the lumen to generate higher shear at the stenosis and low oscillatory shear downstream^26^. Cuff placement for 14 days led to enhanced expression of Snail at the downstream site indicating that low oscillatory shear stress is an activating signal for Snail *in vivo* (Fig. 5).

### Snail was expressed in EC of human atherosclerotic plaques

**Figure 6:**
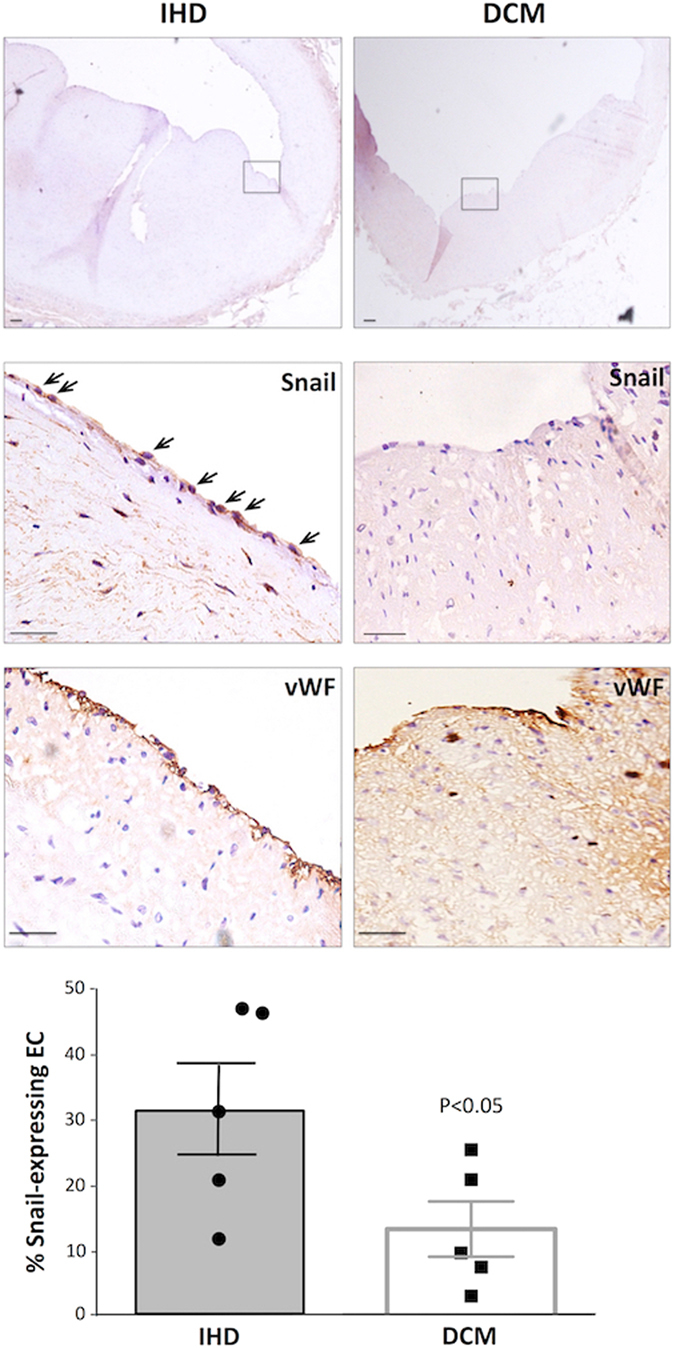
Expression of Snail in endothelium overlying human coronary artery plaques. The expression of Snail in coronary arteries of patients with ischemic heart disease (IHD) or dilated cardiomyopathy (DCM) was studied by immunohistochemistry using specific primary antibodies and HRP-conjugated secondary antibodies (brown). Consecutive sections were stained for EC using anti-vWF antibodies (brown). Sections were counterstained using hematoxylin. Representative images are shown. The region boxed (upper panel, 10X magification) is shown at higher magnifications (centre and lower panels, 40X). Scale bar 20 μm. The proportion of EC that expressed Snail was calculated for each section. Data were pooled from six plaques and mean values +/− SEM are presented. Differences between means were assessed using an unpaired t-test.

Previous studies demonstrated that EC overlying human atherosclerotic plaques express mesenchymal markers^7, 8^. Given our observation that Snail promotes EndMT under low shear stress conditions, we wished to know whether it is involved in human atherosclerosis. Immunohistochemistry of coronary arteries from patients that underwent cardiac transplantation for ischemic heart disease revealed that Snail was expressed in luminal cells overlying coronary artery plaques and co-staining for von Willebrand Factor confirmed that they were of endothelial lineage (Fig. 6). Moreover, the proportion of Snail-positive EC was significantly higher in coronary arteries from patients with ischemic heart disease (advanced atherosclerosis) compared to patients with dilated cardiomyopathy (without symptomatic atherosclerosis; Fig. 6). Thus Snail was detected in EC overlying coronary artery plaques and its expression correlated with advanced atherosclerosis.

## Discussion

### EndMT in atherosclerosis

In addition to its established roles in development^3^, recent studies have revealed that EndMT also contributes to several vascular pathophysiological processes including vascular malformations^4^, pulmonary hypertension^5^ and vascular graft remodelling^6^. Notably several laboratories, including our own, have shown recently that EndMT also contributes to the development of atherosclerosis^7–10^. Chen *et al*.^7^ revealed that EndMT in atherosclerosis is driven by an imbalance in TGF-β and FGFR1 signaling leading to the activation of central regulators of mesenchymal transition including Twist, Snail and Slug. EndMT is also sensitive to local hemodynamics; specifically *in vitro* and *in vivo* studies demonstrated that low shear stress is a positive regulator of EndMT whereas high shear stress is protective^7–9^. The mechanism involved ERK5, which was required for high shear stress-mediated protection^8^ and GATA4-TWIST1 signalling which drives EndMT in response to low shear^9^. In the current study, we further investigated the mechanism that promotes EndMT under low shear stress conditions and identified an essential role for the transcription factor Snail.

Our study demonstrated that Snail was expressed at sites of endogenous low shear stress in healthy murine and porcine arteries, and was induced by the imposition of low shear stress on carotid arteries. Thus we conclude that Snail is expressed at low shear stress regions that are predisposed to atherosclerosis and may therefore contribute to vascular injury and lesion formation. To determine the effects of Snail on EC function and its potential role in vascular dysfunction we carried out gene silencing experiments *in vitro*. This revealed that Snail expression enhanced the permeability to macromolecules in EC exposed to low shear stress. Thus our observation implies that Snail may promote the build-up of cholesterol-rich lipoproteins and other biomolecules in the vessel wall at sites of low shear stress and has obvious implications for the initiation of atherosclerosis, which involves the accumulation of lipoproteins in the intima. Excessive mitosis of EC at atheroprone sites leads to the transient formation of intercellular gaps which enhances the permeability of endothelial monolayers to macromolecules^12, 16^. Thus our observation that Snail positively regulates proliferation of EC provides an explanation for its ability to promote permeability in low shear conditions. Snail also promoted migration of EC exposed to low shear stress, a function that is characteristic of EndMT. Although migration of EC has been documented in the aorta^28^, its relationship to atherosclerosis is uncertain and requires further investigation. Since EndMT has been linked to increased vascular inflammation^9^ it will also be important in future studies to determine the effects of Snail on inflammatory pathways including NF-κB and mitogen-activated protein kinases.

Although it is established that EndMT accompanies atherosclerosis, the extent to which this process contributes to lesion initiation and progression remains uncertain. Evrard *et al*. implicated EndMT in plaque progression because the proportion of endothelial-derived mesenchymal cells in human plaques correlated positively with the severity of disease^10^. However, further work is required to determine the contribution of EndMT to plaque composition and its effects on the physiological and mechanical properties of rupture-prone plaques.

### Reversibility of mechanical EndMT and therapeutic implications

Although it leads to a profound alteration in EC behaviour and appearance, EndMT represents part of a spectrum of EC phenotypes and an example of cellular plasticity^29–31^. EndMT can be reversed in some instances, for example mesenchymal-to-endothelial transition has been described following myocardial infarction^29^. Reversal may be more easily achieved in EC that undergo partial EndMT which involves the activation of mesenchymal genes and simultaneous retention of endothelial marker expression. Partial EndMT has been observed during sprouting angiogenesis in tip cells which lose apical-basal polarity, delaminate and migrate^30^. Although tip cells express markers of EndMT they retain expression of VE-cadherin and other EC markers and continue have contact with neighbouring EC. Because of these characteristics they have been described as undergoing partial EndMT^30^. Similarly, we observed in the current study that low shear stress induced mesenchymal genes whilst retaining expression of VE-cadherin which is consistent with a partial EndMT. The corollary is that atherosclerosis is associated with a partial form of EndMT that may be amenable to reversal towards an endothelial state. This hypothesis should be addressed in future studies to determine whether therapeutic targeting of Snail can treat atherosclerosis by reversing EndMT. Although it is possible that vascular diseases could be treated via EndMT reversal, this is unlikely to be achieved via inhibition of Snail because transcription factors are difficult to target therapeutically. Thus future studies should identify the target genes that are activated by Snail in sheared EC since some of them could provide a therapeutic target to prevent or reverse EndMT.

### Limitations of the study

To assess the role of Snail in EC responses to low shear stress we silenced it by transfecting cells with specific siRNA sequences. It is plausible that the transfection process *per se* can alter cell physiology because it involves electroporation and addition of nucleic acids. Indeed in this study we observed that cells transfected with a non-targeting control siRNA had reduced migration compared to untransfected cells (compare Fig. 2 with Supplementary Fig. 3a), suggesting that siRNA transfection itself can modulate EC function. Nevertheless cell migration under low shear stress was consistently reduced in cells transfected with siRNA targeting Snail compared to control non-targeting sequences. Thus we concluded that silencing of Snail had an effect above and beyond that observed with control sequences indicating that Snail is a positive regulator of migration under low shear conditions.

Another important technical consideration relates to the orbital *in vitro* system that was used to apply flow to cultured EC. Using this system, EC monolayers cultured in 6-well plates were exposed to orbital shaking which generates relatively low shear at the centre of the well and high shear at the periphery. The regions studied were defined previously using computational fluid dynamics to calculate the shear stress distribution over the orbited well^13, 23^. Moreover the altered flow at the centre and periphery was validated by particle imaging velocimetry^13^. Since shear stress is sensitive to changes in the orbital radius of the rotating platform, the frequency of rotation and the viscosity of the cell culture medium, it was ensured that these values were consistent between the current study and those used for computational fluid dynamic calculations^13^. We used a standardized template to identify the regions that were used for EC collection. Nevertheless, the approach is subject to potential sampling errors which could contribute to experimental variation. This was captured by carrying out multiple independent experiments with cells cultured from different individuals. It was observed that low shear stress had consistent effects on several physiological parameters; thus sampling errors were not high enough to mask these biological effects. Finally, since *in vitro* systems do not fully mimic the complex physiology of *in vivo* systems we validated our observations by studying Snail expression in murine, porcine and human arteries.

Although our studies of the aortic arch in pigs and mice demonstrated that Snail expression was enriched in EC located at the inner curvature they did not reveal a causal relationship between shear stress and Snail expression. To address this we modified shear stress in the murine carotid artery using a constrictive cuff developed by Krams^26^. One potential concern with this study is that cuff placement could injure the vessel. However we observed enhanced expression of Snail at a downstream region that was exposed to low oscillatory shear stress but was not exposed to the cuff itself. Another important consideration is the change in shear stress induced by the cuff. This has recently been calculated by Krams who used microCt and ultrasound data to inform computational fluid dynamic simulations^32^. Notably it was found that although shear stress is highly sensitive to geometry there was <10% variation in shear magnitude between cuffed arteries. Similarly, we observed that the expression of Snail at low oscillatory shear stress regions was also consistent between animals and was significantly higher compared to the high shear region or contralateral artery.

## Electronic supplementary material

SUPPLEMENTAL MATERIAL
