# Author Correction: Shear stress induces endothelial-to-mesenchymal transition via the transcription factor Snail

**DOI:** 10.1038/s41598-020-60955-x

**Published:** 2020-02-26

**Authors:** Marwa M. Mahmoud, Jovana Serbanovic-Canic, Shuang Feng, Celine Souilhol, Rouyu Xing, Sarah Hsiao, Akiko Mammoto, Jing Chen, Markus Ariaans, Sheila E. Francis, Kim Van der Heiden, Victoria Ridger, Paul C. Evans

**Affiliations:** 1 Department of Infection, Immunity and Cardiovascular Disease, the INSIGNEO Institute for In Silico Medicine and the Bateson Centre, University of Sheffield, England, UK; 2 ERASMUS MC, Rotterdam, The Netherlands; 3 Vascular Biology Program, Department of Surgery, Boston, MA USA; 4 Department of Ophthalmology, Boston Children’s Hospital, Harvard Medical School, Boston, MA USA

Correction to: *Scientific Reports* 10.1038/s41598-017-03532-z, published online 13 June 2017

The Author Contributions section in this Article is incorrect.

“M.M.M., J.S.-C., S.F., C.S., R.X., S.H., M.A. generated, analysed and interpreted data. A.M., J.C., S.E.F., K.V.D.H., V.R. interpreted data and revised the manuscript for important intellectual content. P.C.E. designed the study, interpreted data and wrote the manuscript.”

should read:

“M.M.M., J.S.-C., S.F., C.S., R.X., S.H., M.A. generated, analysed and interpreted data. A.M., J.S.-C., J.C., S.E.F., K.V.D.H., V.R. interpreted data and revised the manuscript. P.C.E. designed the study, interpreted data and wrote the manuscript.”

