# Supplementary material for: Shear stress induces endothelial-to-mesenchymal transition via the transcription factor Snail
Source: Sci Rep. 2017 Jun 13;7:3375. doi: 10.1038/s41598-017-03532-z (PMC5469771; doi:10.1038/s41598-017-03532-z)
Supplement: Supplementary file 1 — SUPPLEMENTAL MATERIAL [file 41598_2017_3532_MOESM1_ESM.docx]

**SUPPLEMENTAL MATERIAL**

**Shear stress induces endothelial-to-mesenchymal transition via the transcription factor Snail**

Marwa M. Mahmoud, Jovana Serbanovic-Canic, Shuang Feng, Celine Souilhol, Rouyu Xing, Sarah Hsiao, Akiko Mammoto, Jing Chen, Markus Ariaans, Sheila E. Francis, Kim van der Heiden, Victoria Ridger, Paul C. Evans

** Supplementary Figure 1 Analysis of VE-cadherin expression in HUVEC.**

The expression of VE-cadherin was measured to assess the purity of cultured HUVEC. Cells were detached from culture flasks using trypsin and stained using anti-human VE-cadherin antibodies conjugated to PeCy7 or with isotype matched immunoglobulin (IgG1K-PeCy7) as a control. Flourescence was quantified by flow cytometry and is plotted against forward scatter (FSC; a measure of cell size). Representative data from 2 different cords are shown.

** Supplementary Figure 2 Validation of silencing of Snail gene expression.** HUVEC were treated with siRNA targeting Snail or with scrambled non-targeting siRNA. Cells were subsequently cultured in 6 well plates prior to exposure to orbital flow to generate low wall shear stress (WSS) for 72 h. Snail expression was measured by qRT-PCR (a) or by immunofluorescence staining (b).

** Supplementary Figure 3 Baseline studies of EC migration under static or sheared conditions.**

(a) HUVEC were cultured in 6 well plates prior to exposure to high or low WSS for 72 h using an orbital plate system. To assess cell migration, a scratch wound was made in the monolayer, and cells were imaged for 20 h. Representative images are shown (scale bar 100 μm). (b) HUVEC were treated with siRNA targeting Snail, or with scrambled non-targeting siRNA and then cultured under static conditions. To assess cell migration, a scratch wound was made in the monolayer, and cells were imaged for 20 h. The distance migrated at multiple time points (lower left) and average velocity (lower right) was determined. Data were pooled from three independent experiments using cells from different donors and mean levels +/- SEM are shown. Differences between means were assessed using a paired t-test.

** Supplementary Figure 4 Snail did not regulate proliferation in EC exposed to static conditions.**

HUVEC were treated with siRNA targeting Snail or with scrambled (Scr) non-targeting siRNA. Transfected HUVEC were exposed to static conditions. Cell proliferation was quantified by immunofluorescent staining using anti-Ki67 antibodies and co-staining using DAPI. Representative images are shown (Scale bar, 50 μm). The % Ki67-positive cells were calculated for multiple fields of view in three independent experiments using cells from different donors and mean levels +/- SEM are shown. Differences between means were assessed using a paired t-test.

** Supplementary Figure 5 Snail did not regulate permeability in EC exposed to static conditions.**

HUVEC were treated with siRNA targeting Snail or with scrambled (Scr) non-targeting siRNA. They were then cultured on Transwell inserts under static conditions for 72 h prior to assessment of endothelial permeability using rhodamine (Rd)-albumin as a tracer. The concentration of Rd-albumin in the lower compartment was measured and mean values +/- SEM are shown (right panel) from three independent experiments. Differences between means were assessed using a paired t-test.
